# From motivation to movement: autonomous motivation and physical activity levels among students in the Global Health Program

**DOI:** 10.3389/fpsyg.2026.1910169

**Published:** 2026-09-09

**Authors:** O. J. A. Ale, J. Bélisle, I. Doré, N. Carbonneau, T. Barnett, S. Girard, R. F. Guimaraes

**Affiliations:** 1 Department of Physical Activity Sciences, Université du Québec à Trois-Rivières, Trois-Rivières, QC, Canada; 2 School of Kinesiology and Physical Activity Sciences, Faculty of Medicine, Université de Montréal, Montreal, QC, Canada; 3 School of Public Health, Université de Montréal, Montreal, QC, Canada; 4 University of Montreal Hospital Research Centre, Montreal, QC, Canada; 5 Department of Psychology, Université du Québec à Trois-Rivières, Trois-Rivières, QC, Canada; 6 Department of Family Medicine, McGill University, Montreal, QC, Canada; 7 CHU Sainte-Justine Azrieli Research Centre, Montreal, QC, Canada

**Keywords:** adolescents, autonomous motivation, motivational climate, peer affiliation, physical activity, school leaders, school-based intervention, self-determination theory

## Abstract

Little is known about how motivation relates to physical activity within large-scale school-based programs and whether this association varies according to contextual factors such as perceived school leader support, peer affiliation, or duration of program participation. This study examined the association between motivation for physical activity and physical activity levels among students participating in the Global Health Program (GHP) and tested whether this association was moderated by these contextual factors. Cross-sectional data were drawn from the Autumn 2024 baseline evaluation of the GHP, a school-based program implemented across 48 public schools in Québec, Canada. A total of 664 students completed an online questionnaire, with participants represented across 22 schools. Among students with valid age data, the mean age was 14.05 years (SD = 1.80), and 58% were boys. Motivation was assessed using the Behavioural Regulation in Exercise Questionnaire-2, and physical activity was assessed using the PAQ-C or PAQ-A according to school level. Linear mixed-effects models with a random intercept for school were used to examine associations between motivation and physical activity, adjusting for age, sex, and mother’s education. Moderation analyses tested interactions between the Relative Autonomy Index (RAI) and perceived school leader support, peer affiliation, and years of participation. A higher RAI was associated with a higher physical activity score in the adjusted model ( $\hat{\beta }$ = 0.02, 95% CI [0.01, 0.03], *p* < 0.001). Identified regulation ( $\hat{\beta }$ = 0.30, 95% CI [0.24, 0.36], *p* < 0.001) and intrinsic regulation ( $\hat{\beta }$ = 0.28, 95% CI [0.22, 0.35], *p* < 0.001) showed the strongest positive associations with physical activity. Introjected regulation was also positively associated with physical activity ( $\hat{\beta }$ = 0.12, 95% CI [0.08, 0.17], *p* < 0.001), whereas amotivation and external regulation were not statistically significant. Perceived school leader support and peer affiliation did not significantly moderate the association between the RAI and physical activity. However, years of participation significantly moderated this association (interaction $\hat{\beta }$ = 0.004, 95% CI [0.001, 0.008], *p* = 0.022), with a stronger positive association among students reporting longer participation in the GHP. Autonomous motivation was positively associated with physical activity among students participating in the GHP, particularly identified and intrinsic regulation. These findings support the relevance of Self-Determination Theory for understanding physical activity in school-based programs, while longitudinal research is needed to clarify the direction of the observed associations and the role of continued program participation.

## Introduction

Among youth aged 12–17, only about 21% meet the recommendation of at least 60 min of moderate-to-vigorous physical activity per day based on recent Canadian surveillance data (Government of Canada, S. C, 2025). A recent meta-analysis by Tapia-Serrano et al. (2022) further shows that only 7% of young people aged 3 to 18 years meet the 24-Hour Movement Guidelines, which recommend at least 60 min of moderate-to-vigorous intensity physical activity, less than 2 h of recreational screen time, and 9–11 h of sleep per 24-h period (Tremblay et al., 2016). Despite low levels of adherence to these guidelines, it is well established that physical activity provides numerous benefits for both physical and psychosocial health (Carson et al., 2017; Kuzik et al., 2023; Manyanga et al., 2024). Physical activity also supports the development of transferable life skills. Importantly, individuals who have positive experiences with physical activity during childhood are more likely to remain active in adulthood (Miller and Siegel, 2017), highlighting the importance of fostering positive early experiences. In this regard, da Silva et al. (2025) emphasize the importance of a supportive social environments and sustained interventions that encourage youth engagement in physical activity over time.

Within this perspective, the Global Health Program (GHP), implemented among 10,000 students in Québec (Canada), aims to promote sustained behavioural change through school-based educational strategies led by school leaders. These leaders are existing members of the school staff, such as teachers, who receive training and tools to support program implementation. Schools represent an ideal setting for promoting physical activity by engaging not only students, but also their families, peers, and broader communities (Hardie Murphy et al., 2016; Ourda et al., 2025). Previous research highlights the importance of establishing an empowering motivational climate to sustain youth engagement in physical activity both inside and outside school (Blais et al., 2020; Girard et al., 2019, 2023).

In line with these principles, the GHP integrates physical activity into regular class time for all students and relies on trained school leaders who adopt motivational strategies aimed at enhancing students’ motivation and engagement. Although promising, this program requires rigorous evaluation. Indeed, few school-based interventions have been evaluated in real-world settings (Ourda et al., 2025), and those targeting isolated components have often shown limited or no effects (Rodrigo-Sanjoaquín et al., 2025), underscoring the need to better understand why such interventions succeed or fail.

According to Self-Determination Theory (SDT) (Ryan and Deci, 2000), the quality of motivation plays a central role in shaping engagement in physical activity. Motivation lies on a continuum ranging from amotivation to intrinsic motivation, with several forms of extrinsic motivation in between (Ryan and Deci, 2000). At one end of the continuum, amotivation reflects a lack of intention to act. Extrinsic motivation varies in its degree of self-determination and includes external regulation (driven by external rewards or constraints), introjected regulation (internal pressures such as guilt), identified regulation (valuing the behaviour), and integrated regulation (alignment with one’s identity). At the other end of the continuum, intrinsic motivation refers to engaging in an activity for inherent enjoyment, interest, and satisfaction (Ryan and Deci, 2000). Identified and integrated regulations of external motivation are considered more autonomous, alongside intrinsic motivation.

SDT posits that self-determined motivation depends on the satisfaction of three basic psychological needs: autonomy (feeling that one’s actions are self-endorsed and aligned with personal values), competence (feeling effective and capable of mastering tasks), and relatedness (feeling socially connected and supported by others) (Ryan and Deci, 2000). When these needs are satisfied, individuals are more likely to develop autonomous motivation, perform better, and experience greater well-being. Through internalization, motivation can shift along the continuum over time, for example from external regulation (e.g., exercising to lose weight) to intrinsic motivation (e.g., exercising for enjoyment). SDT therefore emphasizes the importance of motivational climates that support autonomy, competence, and relatedness, highlighting that not only the level of motivation, but also its quality, is essential.

To our knowledge, limited research has examined how different forms of motivation relate to physical activity within intervention contexts. Motivational climates can be shaped by school leaders, peers, and the broader social environment (Birr et al., 2023; Deng and Zarrett, 2025; Robinson, 2023; Zhong et al., 2026). When mastery is emphasized over performance, such environments can foster adaptive motivational patterns and contribute to students’ well-being (Duda, 2013; Raimundi et al., 2023).

Recent evidence suggests that school leaders’ positive involvement is associated with students’ motivational quality in physical activity contexts (Guimaraes et al., 2025). School leader implication has been positively linked to autonomous motivation, underscoring their potential role in shaping motivational outcomes (Ntoumanis, 2005).

Therefore, the present study aims to (i) examine whether different forms of motivation for physical activity are associated with physical activity levels among participants in the Global Health Program (GHP), and (ii) test whether these relationships are moderated by perceived school leader implication, peer social network, or duration of program participation. We hypothesize that (i) more autonomous forms of motivation will be associated with higher levels of physical activity, and that (ii) these associations will be stronger among participants reporting greater school leader implication, more supportive peer social networks, and longer participation in the program.

## Materials and methods

### Study design

This cross-sectional study used data collected during the Autumn 2024 baseline assessment of the quasi-experimental evaluation of the Global Health Program (GHP). The GHP is a school-based health promotion program implemented among approximately 10,000 students across 48 public schools in nine administrative regions of Québec, Canada. The program aims to support healthy lifestyle behaviours through two main educational strategies: outdoor-oriented activities and health-related content integrated into school subjects, including nutrition, life balance and stress management, the human body, and first aid. Although the GHP was implemented across 48 schools, students from 22 schools were represented in the present dataset.

### Participants

A total of 664 students completed the baseline questionnaire. All students participating in the GHP were invited to take part in the study through an email distributed by the GHP Director. Participants were recruited from 22 of the 48 public schools in which the program was implemented. Data were collected between September and November 2024 using an online questionnaire administered through Qualtrics and available in French and English. Analyses involving age were restricted to participants with a valid reported age between 6 and 17 years. The number of participants included in each analysis varied according to the availability of complete data for the variables included in the corresponding model.

### Instruments

#### Demographics

Self-reported age, sex at birth, mother’s education, and number of years of participation in the GHP were collected. Age, sex, and mother’s education were included as covariates in the adjusted models. Mother’s education was categorized as secondary education or less, postsecondary or university education, and “do not know.” The number of years of participation in the GHP was examined as a continuous moderator of the association between motivation and physical activity.

#### Motivation

Motivation for physical activity was assessed using the 19-item Behavioural Regulation in Exercise Questionnaire-2 (BREQ-2) (Markland and Tobin, 2004). The questionnaire assesses five motivational regulations along the self-determination continuum: amotivation, external regulation, introjected regulation, identified regulation, and intrinsic regulation. Items were rated on a 5-point Likert scale ranging from 0 (“not true for me”) to 4 (“very true for me”). Mean scores were calculated for each motivational regulation. In the present sample, Cronbach’s alpha coefficients were 0.82 for amotivation, 0.79 for external regulation, 0.71 for introjected regulation, 0.64 for identified regulation, and 0.85 for intrinsic regulation.

The Relative Autonomy Index (RAI) was calculated as follows: (amotivation × − 3) + (external regulation × − 2) + (introjected regulation × − 1) + (identified regulation × 2) + (intrinsic regulation × 3) (Chemolli and Gagné, 2014). The theoretical score ranges from −24 to 20, with higher scores indicating more autonomous motivation toward physical activity. In the present sample, RAI scores ranged from −11 to 19. The RAI was used as the primary motivation measure, while the five individual motivational regulations were examined in separate exploratory analyses.

#### Physical activity

Physical activity during the previous seven days was assessed using the Physical Activity Questionnaire for Older Children (PAQ-C) or the Physical Activity Questionnaire for Adolescents (PAQ-A) (Mestre and Reychler, 2021). The questionnaire version was assigned according to the participant’s reported school level. Students in elementary school grades 1 to 5 completed the PAQ-C scoring structure, whereas students in secondary school grades 1 to 5 completed the PAQ-A scoring structure.

The PAQ-C score was derived from nine components covering physical activity during different periods of the school day, after school, during evenings and weekends, and across the previous week. The PAQ-A included eight corresponding components but excluded the recess item. Each component was scored from 1 to 5, and the final physical activity score was calculated as the mean of the nine PAQ-C components or the eight PAQ-A components. Scores therefore ranged from 1 to 5, with higher scores indicating higher levels of physical activity. Scores were calculated only when all components required for the corresponding questionnaire version were available. In the present sample, Cronbach’s alpha was 0.79 for the PAQ-C and 0.78 for the PAQ-A.

#### Students’ perception of school leaders’ implication

Students’ perceptions of school leader support within the GHP were assessed using a seven-item researcher-developed scale. The items were developed by the research team in collaboration with the GHP Director and assessed whether the school leader created an enjoyable atmosphere, encouraged participation, proposed safe activities adapted to students’ abilities, was open to dialogue, promoted respect among peers, supported a sense of belonging, and inspired students to engage in physical activity and healthy lifestyle behaviours. Items were rated on a 5-point Likert scale ranging from 1 (“strongly disagree”) to 5 (“strongly agree”).

A mean score was calculated when at least five of the seven items were completed, with higher scores indicating greater perceived school leader support. The scale demonstrated excellent internal consistency in the present sample (Cronbach’s α = 0.93). For descriptive analyses only, the mean score was categorized as positive perceived support (mean score ≥ 4) or less positive perceived support (mean score < 4). The continuous score was used in the moderation analyses.

#### Peer involvement in the GHP

Peer affiliation within the GHP was assessed using the question: “Are your best friends students from your school who participate in the Global Health Program?” Response options were “all of my friends,” “some of them,” “almost none,” and “none.” Responses were dichotomized as having best friends in the GHP (“all” or “some”) versus not having best friends in the GHP (“almost none” or “none”). This variable was examined as a potential moderator of the association between the RAI and physical activity.

## Data management

Data were examined for completeness and consistency before analysis. Age values outside the eligible range of 6 to 17 years were treated as missing in analyses involving age. Mother’s education was categorized as secondary education or less, postsecondary or university education, and “do not know”; only truly missing responses were coded as missing. The PAQ-C and PAQ-A scores were calculated only when all components required for the relevant questionnaire version were available. The school leader support score was calculated when at least five of the seven items were completed.

For descriptive purposes only, PAQ scores were categorized as inactive (1.00–1.99), very little active (2.00–2.99), active (3.00–3.99), or very active (4.00–5.00), and subsequently collapsed as inactive versus active. These categories were used to describe the distribution of PAQ scores and were not interpreted as indicating adherence to national physical activity guidelines. Continuous predictors involved in interaction terms were mean-centered before analysis. Analyses were conducted using complete cases for the variables included in each specific model.

## Statistical analyses

Continuous variables were summarized using means and standard deviations, while categorical variables were summarized using frequencies and percentages. Internal consistency was evaluated using Cronbach’s alpha for each BREQ-2 subscale, the PAQ-C, the PAQ-A, and the school leader support scale.

Because participants were nested within schools, the potential clustering of physical activity scores at the school level was first examined using an intercept-only linear mixed-effects model. The intraclass correlation coefficient was 0.05, indicating that approximately 5% of the variance in PAQ scores occurred between schools. Consequently, linear mixed-effects models with a random intercept for school were used for all association and moderation analyses.

The association between the RAI and physical activity score was examined using a crude mixed-effects model and a model adjusted for age, sex, and mother’s education. Separate adjusted mixed-effects models were then estimated for amotivation, external regulation, introjected regulation, identified regulation, and intrinsic regulation. These models were conducted to examine the association between each motivational regulation and physical activity.

Moderation analyses examined whether the association between the centered RAI and physical activity score varied according to perceived school leader support, peer affiliation within the GHP, or number of years of participation in the program. Each moderator and its interaction with the RAI were examined in a separate mixed-effects model adjusted for age, sex, and mother’s education and including a random intercept for school. When a statistically significant interaction was identified, simple slopes were estimated at the mean number of years of participation and at one standard deviation below and above the mean.

Model assumptions were evaluated using residual and influence diagnostics. Multicollinearity was assessed using variance inflation factors, homoscedasticity was examined using residual diagnostics, and influential observations were assessed using Cook’s distance. Residual normality was evaluated using formal testing and visual inspection of Q-Q plots. Marginal and conditional R^2^ values were calculated to estimate the variance explained by the fixed effects and by the complete mixed-effects model, respectively. Regression coefficients, 95% confidence intervals, and *p*-values were reported. Statistical significance was set at *p* < 0.05. Analyses were conducted in R version 4.5.1 using complete cases for the variables included in each model (the Comprehensive R Archive Network, s. d.) (R Core Team, R Foundation for Statistical Computing, Vienna, Austria).

## Results

A total of 664 students completed the baseline questionnaire, with participants represented across 22 schools (Table 1). Among participants with a valid age between 6 and 17 years, the mean age was 14.05 years (SD = 1.80). Girls represented 42.0% of participants and boys 58.0%. Regarding mother’s education, 9.4% reported secondary education or less, 72.3% reported postsecondary or university education, and 18.3% selected “do not know.” The mean duration of participation in the GHP was 3.07 years (SD = 2.11), and approximately half of participants with available data had participated for 3–4 years (51%).

**Table 1:** Characteristics of study participants (*n* = 664).

Characteristic	*N* = 664
**Age, mean (SD)**	14.05 (1.80)
Sex, *n* (%)	
Girls	277 (42%)
Boys	383 (58%)
Mother’s education, *n* (%)	
≤ secondary	62 (9.4%)
Postsecondary / university	475 (72.3%)
Do not know	120 (18.3%)
**Years of participation in GHP, mean (SD)**	3.07 (2.11)
Years of participation in GHP, *n* (%)	
1–2 years	152 (26.5%)
3–4 years	292 (51%)
≥5 years	129 (22.5%)
**PAQ score, mean (SD)**	2.85 (0.58)
Physical activity level, *n* (%)	
Inactive	377 (61%)
Active	241 (39%)
**RAI, mean (SD)**	11.93 (4.90)
**School leader support, mean (SD)**	3.87 (0.82)
School leader support, *n* (%)	
Less positive support	282 (48.7%)
Positive support	297 (51.3%)
Peers support, *n* (%)	
No friends in the GHP	57 (9.5%)
Best friends in the GHP	540 (90.5%)

GHP, Global Health Program; PAQ, Physical Activity Questionnaire; RAI, Relative Autonomy Index; SD, standard deviation. Data are presented in means (SD) or *n* (%). * PAQ-C and PAQ-A questionnaire. Scores are interpreted as follows: 1 and 2 = Inactive; 3 and 4 = Active; 5 = Very active. ×Likert scale from 1 (strongly disagree) to 5 (strongly agree).

The mean physical activity score was 2.85 (SD = 0.58). Among participants with a valid PAQ score, 39% were descriptively classified as active and 61.0% as inactive. The mean RAI score was 11.93 (SD = 4.90). The mean perceived school leader support score was 3.87 (SD = 0.82), with 51.3% categorized as reporting positive support and 48.7% as reporting less positive support. Most participants with available peer-affiliation data reported having best friends participating in the GHP (90.5%), while 9.5% reported having no best friends in the program.

### Association between motivation and physical activity

The association between the RAI and physical activity score is presented in Table 2. In the crude linear mixed-effects model, a higher RAI was associated with a higher physical activity score ( $\hat{\beta }$ = 0.02, 95% CI [0.01, 0.03], *p* < 0.001). This association remained statistically significant after adjustment for age, sex, and mother’s education ( $\hat{\beta }$ = 0.02, 95% CI [0.01, 0.03], *p* < 0.001). Age was not statistically significantly associated with physical activity score ( $\hat{\beta }$ = − 0.02, 95% CI [−0.05, 0.01], *p* = 0.20). Boys had higher physical activity scores than girls ( $\hat{\beta }$ = 0.25, 95% CI [0.16, 0.34], *p* < 0.001). Compared with participants reporting maternal education at the secondary level or below, those reporting postsecondary or university education did not differ significantly in physical activity score ( $\hat{\beta }$ = − 0.05, 95% CI [−0.21, 0.11], *p* = 0.50). Participants who selected “do not know” for mother’s education had a lower physical activity score ( $\hat{\beta }$ = − 0.20, 95% CI [−0.38, −0.01], *p* = 0.035). This latter coefficient should be interpreted cautiously because the “do not know” category does not represent a defined level of maternal education.

**Table 2:** Association between relative autonomy index and physical activity score.

Variable	Model 1: Crude			Model 2: Adjusted		
	$\hat{\beta }$	95% CI	*p*-value	$\hat{\beta }$	95% CI	*p*-value
RAI	0.02	0.01, 0.03	<0.001	0.02	0.01, 0.03	<0.001
Age				−0.02	−0.05, 0.01	0.2
Sex						
Girls				Reference		
Boys				0.25	0.16, 0.34	<0.001
Mother’s education						
≤ secondary				Reference		
Postsecondary / university				−0.05	−0.21, 0.11	0.5
Do not know				−0.20	−0.38, −0.01	0.035

RAI: Relative Autonomy Index; CI: confidence interval. Estimates were obtained from linear mixed-effects models with a random intercept for school. Both models included 596 participants.

**Figure 1:**
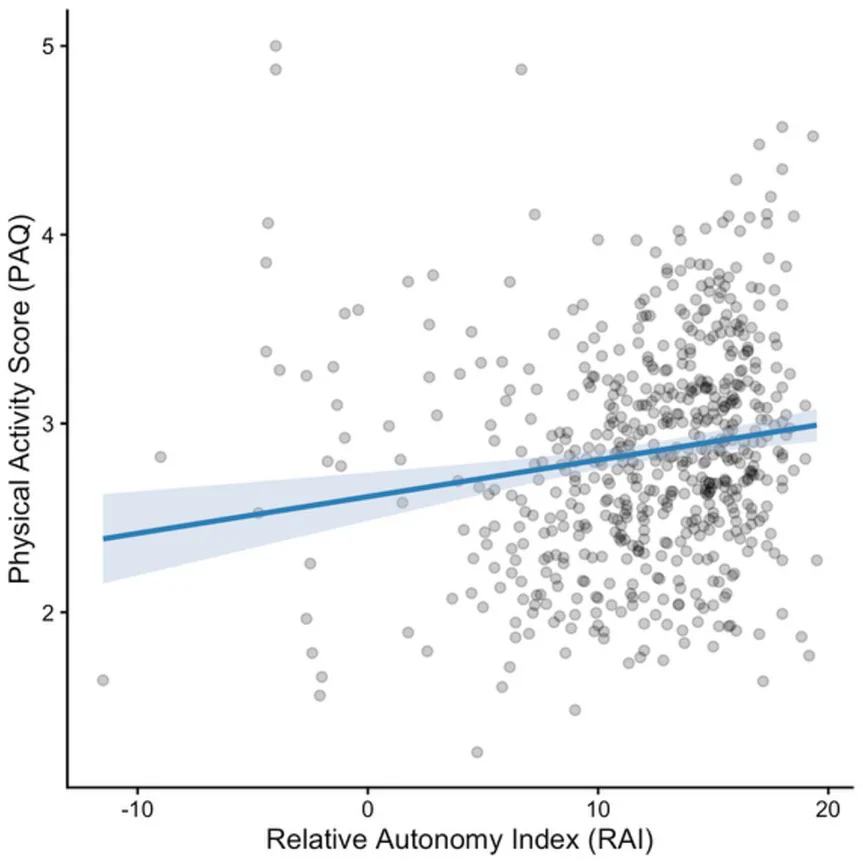
This figure presents the unadjusted descriptive association between the RAI and physical activity score.

Model diagnostics indicated low multicollinearity, with all variance inflation factors close to 1, homoscedastic residuals, and no influential observations. Although the formal test of residual normality was statistically significant, visual inspection of the Q-Q plot indicated only modest deviations in the distribution tails. The adjusted model had a marginal R^2^ of 0.084 and a conditional R^2^ of 0.127 (see Figure 1).

### Types of motivation and physical activity

**Figure 2:**
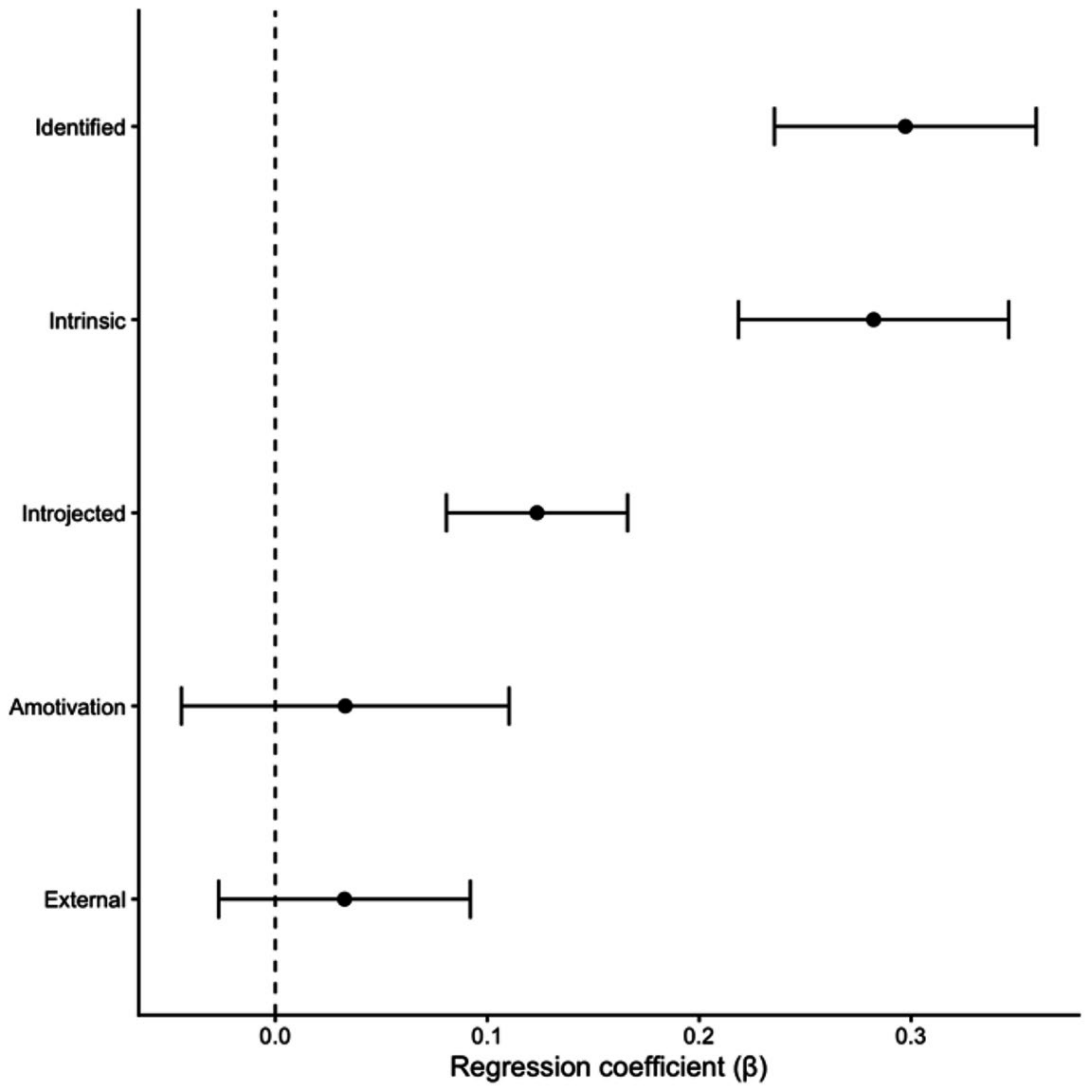
Adjusted associations between motivational regulations and physical activity score.

The associations between the different motivational regulations and physical activity scores are presented in Table 3 and Figure 2. Identified regulation showed the strongest positive association with physical activity score ( $\hat{\beta }$ = 0.30, 95% CI [0.24, 0.36], *p* < 0.001), followed by intrinsic regulation ( $\hat{\beta }$ = 0.28, 95% CI [0.22, 0.35], *p* < 0.001). Introjected regulation was also positively associated with physical activity, although the magnitude of the association was smaller ( $\hat{\beta }$ = 0.12, 95% CI [0.08, 0.17], *p* < 0.001). In contrast, amotivation ( $\hat{\beta }$ = 0.03, 95% CI [−0.04, 0.11], *p* = 0.40) and external regulation ( $\hat{\beta }$ = 0.03, 95% CI [−0.03, 0.09], *p* = 0.30) were not statistically significantly associated with physical activity score. Each motivational regulation was examined in a separate linear mixed-effects model adjusted for age, sex, and mother’s education, with a random intercept for school.

**Table 3:** Association between types of motivation and physical activity score.

Type of motivation	$\hat{\beta }$	95% CI	*p*-value
Amotivation	0.03	−0.04, 0.11	0.40
External regulation	0.03	−0.03, 0.09	0.30
Introjected regulation	0.12	0.08, 0.17	<0.001
Identified regulation	0.30	0.24, 0.36	<0.001
Intrinsic regulation	0.28	0.22, 0.35	<0.001

Each motivational regulation was examined in a separate linear mixed-effects model adjusted for age, sex, and mother’s education, with a random intercept for school. Model sample sizes ranged from 594 to 596 participants. CI, confidence interval.

### Moderation analyses

**Figure 3:**
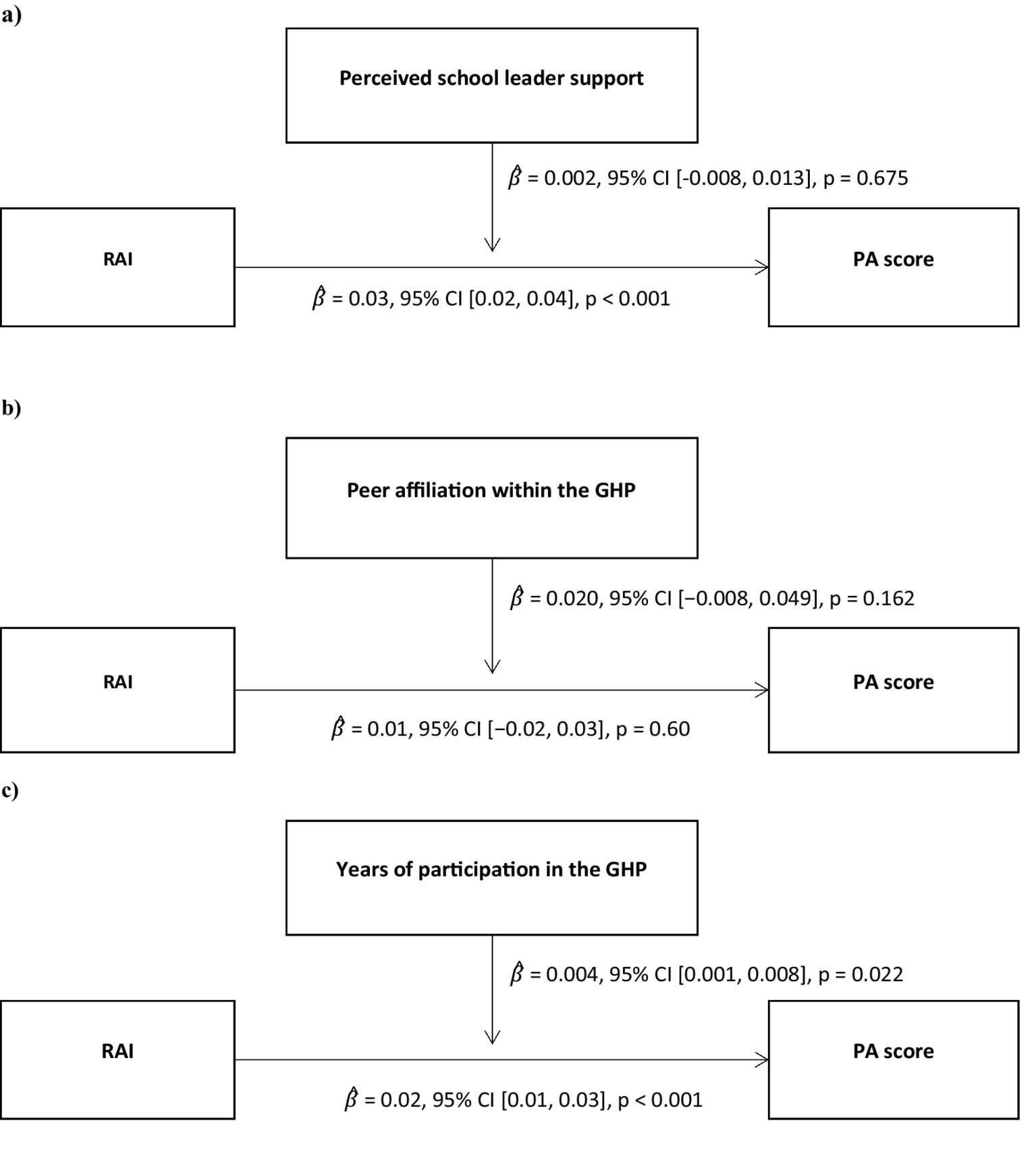
**(a)** Perceived school leader support. **(b)** Peer affiliation within the GHP. **(c)** Years of participation in the GHP.

The moderation analyses are presented in Figure 3. Perceived school leader support did not significantly moderate the association between the RAI and physical activity score (interaction $\hat{\beta }$ = 0.002, 95% CI [−0.008, 0.013], *p* = 0.675). Peer affiliation within the GHP was also not a statistically significant moderator of this association (interaction $\hat{\beta }$ = 0.020, 95% CI [−0.008, 0.049], *p* = 0.162).

In contrast, the number of years of participation in the GHP significantly moderated the association between the RAI and physical activity score (interaction $\hat{\beta }$ = 0.004, 95% CI [0.001, 0.008], *p* = 0.022). Simple-slope analyses indicated that the association was not statistically significant at approximately 1 year of participation ( $\hat{\beta }$ = 0.011, 95% CI [−0.002, 0.024]), but was positive at approximately 3 years of participation ( $\hat{\beta }$ = 0.020, 95% CI [0.010, 0.029]) and stronger at approximately 5 years of participation ( $\hat{\beta }$ = 0.029, 95% CI [0.017, 0.041]). These findings indicate that the positive association between autonomous motivation and physical activity was stronger among students reporting longer participation in the GHP.

## Discussion

This study examined the association between motivation for physical activity and physical activity levels among students participating in the GHP, while accounting for the clustering of students within schools. Overall autonomous motivation, measured using the RAI, was positively associated with physical activity score after adjustment for age, sex, and mother’s education. When the motivational regulations were examined separately, identified and intrinsic regulations showed the strongest positive associations with physical activity, while introjected regulation was also positively associated with physical activity. Amotivation and external regulation were not statistically significantly associated with physical activity. Perceived school leader support and peer affiliation did not moderate the association between the RAI and physical activity. However, the association was stronger among students reporting a longer duration of participation in the GHP. Given the cross-sectional design, these results describe associations and do not establish their direction or causality.

### Sex and age differences

Boys reported higher physical activity scores than girls, a finding consistent with international evidence documenting lower physical activity levels among adolescent girls. In contrast, age was not statistically significantly associated with physical activity in the primary adjusted model, although the estimated coefficient was negative. A multilevel study conducted in physical education settings similarly found that boys tended to report higher autonomous motivation and lower amotivation and that more physically active students showed higher autonomous motivation (Sánchez-Oliva et al., 2020).

Previous interventions emphasizing supportive school leader behaviours and inclusive pedagogical approaches have shown potential for improving girls’ intrinsic motivation (Barbosa Cano and Gomez-Baya, 2025). Although the present study did not examine sex-specific motivational pathways, the lower physical activity scores observed among girls suggest that school-based programs may need to consider how activities, choices, feedback, and social environments are experienced by students of different sexes. Future studies could investigate whether the associations between motivational regulations and physical activity vary by sex.

### Autonomous motivation and physical activity

Our findings complement a substantial body of research showing that more autonomous reasons for being active are positively associated with physical activity behaviour. A systematic review found that intrinsic and identified regulations, which represent more self-determined forms of motivation, were associated with persistent physical activity and exercise behaviour, whereas more controlled forms of motivation tended to be more closely related to short-term participation (Teixeira et al., 2012). Identified regulation, which reflects the personal value attributed to physical activity, may be particularly relevant to the adoption of activity, whereas intrinsic motivation may be more closely related to longer-term adherence (Rachele et al., 2015).

The strong associations observed for identified and intrinsic regulations are consistent with the theoretical predictions of SDT. The framework distinguishes intrinsic and identified regulations as more autonomous forms of motivation, while introjected and external regulations are considered more controlled (Ryan and Deci, 2000; Teixeira et al., 2012). Autonomous motivation has been associated with enjoyment, vitality, and sustained engagement, whereas controlled motivation may involve internal pressure, guilt, or external demands and has been associated with less adaptive experiences such as boredom (White et al., 2021). Our findings are also consistent with multilevel analyses conducted in physical education settings, in which intrinsic and identified motivation were positively associated with intention and effort, whereas external regulation and amotivation were not (Sánchez-Oliva et al., 2020).

Introjected regulation was also positively associated with physical activity in the present study, although its association was smaller than those observed for identified and intrinsic regulation. This may indicate that internal pressures, such as guilt or self-imposed expectations, can be associated with participation in physical activity without necessarily representing the most adaptive or sustainable motivational basis for engagement. Because the present study was cross-sectional, it cannot determine whether motivational regulation influences physical activity, whether physical activity experiences shape motivation, or whether the association operates in both directions.

The present findings are consistent with SDT, which proposes that satisfaction of the needs for autonomy, competence, and relatedness is associated with the development of more autonomous motivation. Within the GHP, activities that allow students to experience competence, make meaningful choices, and interact positively with peers and school leaders may support the internalization of physical activity behaviours and may be associated with greater engagement.

### Social environment and autonomy-supportive contexts

Perceived school leader support and peer affiliation did not significantly moderate the association between autonomous motivation and physical activity. Therefore, the present findings do not provide evidence that the relationship between the RAI and physical activity differed according to these two contextual factors. This does not demonstrate that school leaders and peers are unimportant. Rather, these factors may operate through mechanisms other than moderation, such as contributing to need satisfaction, influencing the development of motivation, or being directly associated with engagement. These possible pathways were not tested in the present study.

Evidence from previous research indicates that autonomy-supportive contexts, including those that provide choice, rationale, positive feedback, and opportunities for competence and relatedness, are associated with more self-determined motivation and physical activity (White et al., 2021). A systematic review of SDT in physical education similarly found that supportive teacher behaviours were positively associated with need satisfaction and intrinsic motivation, whereas controlling behaviours were associated with amotivation and negative affect (Vasconcellos et al., 2020). These findings provide a theoretical basis for continuing to train school leaders in need-supportive practices. However, because school leader support did not moderate the association between motivation and physical activity in the present study, its role should not be interpreted as modifying the behavioural relevance of autonomous motivation.

A previous study using GHP data reported that greater perceived school leader involvement was associated with more autonomous motivation and higher physical activity (Guimaraes et al., 2025). Considered alongside the present findings, this evidence is compatible with the possibility that school leaders contribute to the motivational context in which students participate. Nevertheless, the present moderation results indicate that perceived school leader support did not change the strength of the association between the RAI and physical activity. Future longitudinal or mediation analyses would be better suited to examining whether school leader support contributes to physical activity indirectly through autonomous motivation.

### Years of participation to GHP

In contrast, years of participation significantly moderated the association between autonomous motivation and physical activity. The association was not statistically significant at approximately one year of participation but was positive at approximately three years and stronger at approximately five years. Although few studies have directly examined duration of program participation as a moderator of the association between motivation and physical activity, longitudinal evidence suggests that motivational processes and physical activity may evolve together during continued exposure to supportive activity contexts. Deng et al. (2023) found that changes in intrinsic motivation were positively associated with changes in adolescents’ moderate-to-vigorous physical activity over a 16-week after-school intervention. Similarly, a longitudinal study of primary school students found that autonomously motivated profiles were associated with more adaptive physical activity outcomes over time than amotivated or predominantly controlled profiles (Tapia-Serrano et al., 2024).

One possible interpretation of the present finding is that longer participation may provide repeated opportunities for students to experience enjoyment, competence, social connection, and personally meaningful reasons for being active. Such repeated experiences could make the association between autonomous motivation and physical activity more apparent among students with longer exposure to the program. However, the present cross-sectional design does not establish that continued participation strengthened this association. Students with greater autonomous motivation or higher physical activity may also have remained in the program longer, and years of participation may reflect differences in previous exposure, school context, or other unmeasured characteristics. Longitudinal analyses are therefore needed to determine whether changes in motivation and physical activity become more closely related as participation in the GHP continues (Courtney et al., 2021).

### Implications for practice

The positive associations observed for identified and intrinsic regulation suggest that school-based physical activity programs should consider both enjoyment and personal value when designing activities. Consistent with SDT and previous evidence, programs may support autonomy, competence, and relatedness by providing meaningful choices, offering an understandable rationale for activities, adapting tasks to students’ abilities, and encouraging respectful and supportive interactions (Ryan and Deci, 2000; Teixeira et al., 2012). These strategies may contribute to motivational environments in which students are more likely to report autonomous reasons for being active.

Although perceived school leader support and peer affiliation did not moderate the association between motivation and physical activity, both remain relevant components of the broader motivational environment described in previous research (Deng and Zarrett, 2025; Robinson, 2023; Zhong et al., 2026). The present findings do not establish that increasing school leader support or peer affiliation will increase physical activity, but they support further investigation of whether these contextual factors influence the development of motivation through indirect pathways. The significant interaction with years of participation also suggests that motivational processes may differ according to the duration of students’ involvement in the program. Programs may therefore benefit from considering how motivational support is maintained and adapted as students continue their participation over several years.

While intrinsic motivation represents the most self-determined form of motivation, identified regulation may also be an important pathway for engaging young people in physical activity when they recognize its personal value and relevance (O’Dougherty et al., 2010). Interventions may therefore benefit from combining enjoyable activities with age-appropriate explanations of how physical activity relates to health, well-being, social connection, and daily functioning. Such strategies may support the internalization of physical activity behaviours, although longitudinal studies are required to determine whether they contribute to sustained participation over time.

## Strengths and limitations

This study has several strengths. It included a relatively large sample of students participating in a real-world school-based program and represented 22 public schools across Québec. The analyses examined both the overall RAI and the individual motivational regulations, used established measures of motivation and physical activity, and accounted for the clustering of students within schools. The study also examined contextual moderators that have received limited attention in large-scale school-based intervention settings. The researcher-developed school leader support scale demonstrated excellent internal consistency in the present sample.

Some limitations should nevertheless be considered. First, the cross-sectional design prevents conclusions regarding temporality or causality, and reverse or bidirectional associations between motivation and physical activity are possible. Second, physical activity, motivation, school leader support, and peer affiliation were all self-reported, which may have introduced recall, social desirability, and common-method bias. Third, although models were adjusted for age, sex, and mother’s education, residual confounding may remain because socioeconomic circumstances, parental physical activity, access to recreational opportunities, and psychological factors such as self-efficacy were not measured (de Oliveira Barbosa et al., 2024; Hosokawa et al., 2023). In addition, integrated regulation was not assessed, limiting examination of the full self-determination continuum (Slawinski et al., 2020). Fourth, the high proportion of participants reporting postsecondary or university maternal education, together with the number selecting “do not know,” may also limit the socioeconomic representativeness of the sample. Finally, participants were drawn exclusively from the GHP in Québec, and the findings may not generalize to students outside this program or to other educational and cultural settings.

## Conclusion

Greater autonomous motivation was associated with higher physical activity scores among students participating in the GHP, particularly for identified and intrinsic regulation. Introjected regulation was also positively associated with physical activity, whereas amotivation and external regulation were not. Perceived school leader support and peer affiliation did not modify the association between autonomous motivation and physical activity, while this association was stronger among students reporting longer participation in the program.

These findings support the relevance of SDT for understanding physical activity within school-based programs and suggest that both enjoyment and the personal value attributed to physical activity should be considered when designing motivationally supportive environments. However, longitudinal research is needed to determine the direction of these associations and to examine whether continued participation in the GHP contributes to changes in motivation and physical activity over time.

## Data Availability

The raw data supporting the conclusions of this article will be made available by the authors, without undue reservation.
